# Potential mechanism prediction of Cold-Damp Plague Formula against COVID-19 via network pharmacology analysis and molecular docking

**DOI:** 10.1186/s13020-020-00360-8

**Published:** 2020-07-30

**Authors:** Lin Han, Xiu-Xiu Wei, Yu-Jiao Zheng, Li-Li Zhang, Xin-Miao Wang, Hao-Yu Yang, Xu Ma, Lin-Hua Zhao, Xiao-Lin Tong

**Affiliations:** 1 Guang’anmen Hospital, China Academy of Chinese Medical Sciences, Beijing, 100053 China; 2 Beijing University of Chinese Medicine, Beijing, 100029 China; 3 Gansu University of Chinese Medicine, Lanzhou, 730000 China

**Keywords:** COVID-19, Cold‒Damp Plague Formula (CDPF), Network pharmacology, Molecular mechanism, Molecular docking

## Abstract

**Background:**

Coronavirus disease 2019 (COVID-19) is a new global public health emergency. The therapeutic benefits of Cold‒Damp Plague Formula (CDPF) against COVID-19, which was used to treat “cold‒dampness stagnation in the lung” in Trial Versions 6 and 7 of the “Diagnosis and Treatment Protocol for COVID-19”, have been demonstrated, but the effective components and their mechanism of action remain unclear.

**Methods:**

In this study, a network pharmacology approach was employed, including drug-likeness evaluation, oral bioavailability prediction, protein‒protein interaction (PPI) network construction and analysis, Gene Ontology (GO) terms, and Kyoto Encyclopedia of Genes and Genomes (KEGG) pathway annotation, and virtual docking, to predict the bioactive components, potential targets, and molecular mechanism of CDPF for COVID-19 treatment.

**Results:**

The active compound of herbs in CDPF and their candidate targets were obtained through database mining, and an herbs—ingredients—targets network was constructed. Subsequently, the candidate targets of the active compounds were compared to those relevant to COVID-19, to identify the potential targets of CDPF for COVID-19 treatment. Subsequently, the PPI network was constructed, which provided a basis for cluster analysis and hub gene screening. The seed targets in the most significant module were selected for further functional annotation. GO enrichment analysis identified four main areas: (1) cellular responses to external stimuli, (2) regulation of blood production and circulation, (3) free radical regulation, (4) immune regulation and anti-inflammatory effects. KEGG pathway analysis also revealed that CDPF could play pharmacological roles against COVID-19 through “multi components‒multi targets‒multi pathways” at the molecular level, mainly involving anti-viral, immune-regulatory, and anti-inflammatory pathways; consequently, a “CDPF—herbs—ingredients—targets—pathways—COVID-19” network was constructed. In hub target analysis, the top hub target IL6, and ACE2, the receptor via which SARS-CoV-2 typically enters host cells, were selected for molecular docking analyses, and revealed good binding activities.

**Conclusions:**

This study revealed the active ingredients and potential molecular mechanism by which CDPF treatment is effective against COVID-19, and provides a reference basis for the wider application and further mechanistic investigations of CDPF in the fight against COVID-19.

## Background

Novel coronavirus pneumonia (NCP) is an infectious disease induced by severe acute respiratory syndrome coronavirus 2 (SARS-CoV-2), which is characterized by a long incubation period, rapid spread, and general population susceptibility. The disease may progress to pneumonia, acute respiratory distress syndrome, acute kidney injury, shock, and multi organ dysfunction [1]. Since January 2020, the virus spread rapidly to most parts of China as well as to other countries worldwide, and the World Health Organization (WHO) declared the disease outbreak as the sixth public health emergency of international concern on January 30, 2020 [2]. NCP was officially named coronavirus disease 2019 (COVID-19) by the WHO on February 11, 2020 [3]. As of May 1, 2020, data released by the WHO revealed 3,175,207 known cases of COVID-19, 224,172 reported deaths, and 211 affected countries or regions [4], these figures are updated daily and are expected to increase further. COVID-19 poses a marked threat to the safety and health of the world’s population, and the global impact of the outbreak continues to expand. As no specific drug or vaccine against COVID-19 has been identified to date, creation of a rapid and effective prevention and control program, curbing the spread of disease, and reducing the damage caused by the pandemic are major challenges.

In its long history, Traditional Chinese Medicine (TCM) has accumulated a solid theoretical basis for the treatment of infectious diseases [5], and has recently been implemented against viral pneumonia. TCM have been reported to enhance the immunity of the organism, shorten the duration of treatment, and prevent complications according to numerous studies [6–10]. TCM showed a good treatment effect against severe acute respiratory syndrome (SARS) in 2003. Compared with pure Western medicine-treatment, treatment with pure Chinese herbal medicine could significantly shorten the average absorption time of the lung shadow, without any obvious complications in follow-up, in SARS patients [7]. In the COVID-19 epidemic, China has used a combination of Chinese and Western medicine for both the prevention and treatment of the disease, with notable positive effects. With the strong support of the Chinese government, TCM has been used in the treatment of 91.5% of confirmed cases; inclusion of TCM in COVID-19 treatment not only reduced the death rate of severe/critical patients by more than 80%, but also resulted in a low rate of relapse in recovered patients; the total effective rate of TCM is more than 90% [11, 12]. Further, more and more studies have analyzed mechanisms of action of TCM formulae in treating COVID-19 [13–17]. TCM has been recommended for different stages of COVID-19 treatment since the third trial version of the “Diagnosis and Treatment Protocol for COVID-19” published by National Health Commission of the People’s Republic of China, along with Western standard treatment [14, 18].

In trial versions 6 and 7 of the “Diagnosis and Treatment Protocol for COVID-19,” the Cold‒Damp Plague Formula (CDPF) was recommended for treating “cold‒dampness stagnation in the lung,” as the disease was designated as a “cold‒dampness pestilence” based on the clinical features of COVID-19 cases in Wuhan [19]. The formula was devised by academician Xiao-Lin Tong and other experts, particularly for use in suspected and early-stage confirmed COVID-19 cases, and was mainly used in the epicentre of the epidemic, Wuhan, as well as in other large epidemic areas [19–21]. CDPF, consisting of 20 herbs (Table 1), is based on several classical prescriptions, including Ma Xing Shi Gan decoction, Ting Li Da Zao Xie Fei decoction, Huo Po Xia Ling decoction, Shen Zhu San, and Da Yuan Yin, and has the TCM functions of ventilating the lungs and expelling pathogenic factors, dispelling toxins and dredging collaterals, dissolving turbidity, strengthening the spleen, and eliminating dampness [19]. More than 50,000 COVID-19 patients, including mild cases, moderate cases, suspected cases, and self-isolating fever cases, have taken CDPF, which showed significant advantages in improving the cure rate, reducing mortality, and promoting physical recovery [12, 22].

**Table 1:** The composition of herbs in CDPF

Botanical name	English name	Chinese name	Abbreviation
*Herba Ephedrae*	Ephedra	Ma Huang	MH
*Semen Armeniacae Amarum*	Bitter apricot seed	Ku Xing Ren	KXR
*Gypsum Fibrosum*	Gypsum	Shi Gao	SG
*Rhizoma et Radix Notopterygii*	Incised notopterygium rhizome and root	Qiang Huo	QH
*Semen Descurainiae*	Semen lepidii	Ting Li Zi	TLZ
*Rhizoma Dryopteris Crassirhizomae*	Male fern rhizome	Guan Zhong	GZ
*Radix Cynanchi Paniculati*	Paniculate swallowwort root	Xu Chang Qing	XCQ
*Herba Pogostemonis*	Cablin patchouli herb	Guang Huo Xiang	GHX
*Herba Eupatorii*	Fortune eupatorium herb	Pei Lan	PL
*Rhizoma Atractylodis*	Atractylodes rhizome	Cang Zhu	CZ
*Poria*	Indian bread	Fu Ling	FL
*Rhizoma Atractylodis Macrocephalae*	Largehead atractylodes rhizome	Bai Zhu	BZ
*Cortex Magnoliae Officinalis*	Officinal magnolia bark	Hou Po	HP
*Fructus Tsaoko*	Fruit of caoguo	Cao Guo	CG
*Lumbricus*	Pheretima	Di Long	DL
*Fructus Crataegi*	Charred hawthorn	Jiao Shan Zha	JSZ
*Massa Medicata Fermentata*	Fried medicated leaven	Jiao Shen Qu	JSQ
*Fructus Hordei Germinatus*	Burnt malt	Jiao Mai Ya	JMY
*Semen Arecae*	Arecaesementostum	Jiao Bing Lang	JBL
*Rhizoma Zingiberis Recens*	Fresh ginger	Sheng Jiang	SJ

The effect of TCM is related to the mechanism of the multi-ingredients, multi-targets, and multi-pathways of herbs; however, this multi-faceted mechanism makes it difficult to identify the mechanism of action of CDPF in terms of traditional pharmacological evaluation. On the other hand, network pharmacology is an effective approach to study and clarify the mechanism of drug action, which could explain the mechanism of functional drugs based on a network of public databases or data obtained through early studies. The network pharmacology research strategy is in line with the understanding of disease integrity in TCM [23, 24].

Thus, in this study, we utilized network pharmacology and the related technology to explore the main bioactive components of CDPF and predict the effective targets of these active components. Consequently, we proposed potential mechanisms underlying the effects of CDPF in the treatment of COVID-19 at a molecular level, which might provide theoretical support for the wider application of CDPF in the fight against COVID-19.

## Methods

### Collection of ingredients of CDPF and screening

The components of herbs used in CDPF were retrieved from the Traditional Chinese Medicine Systems Pharmacology (TCMSP**)** database (http://tcmspw.com/tcmsp.php) and Traditional Chinese Medicines Integrated Database (TCMID) (http://www.megabionet.org/tcmid/) [25], and related references. The candidate ingredients from the TCMSP were retained only if their oral bioavailability (OB) ≥ 30% and if their drug likeness (DL) ≥ 0.18 [26]. Candidate ingredients without potential target information were excluded. The properties of ingredients collected through the TCMID and literature reviews were retrieved from the Swiss ADME database (http://www.swissadme.ch/). The screening criterion for gastrointestinal GI absorption was set as high, and DL is satisfied with both “yes” at the same time [27]. The structures and SMILES format files of the compounds were obtained from the PubChem (https://pubchem.ncbi.nlm.nih.gov/) database [28].

### Construction of targets related to the identified compounds

To obtain the target of each identified compound, putative targets were predicted from three databases: the TCMSP, similarity ensemble approach (SEA) (http://sea.bkslab.org) [29], and STITCH (http://stitch.embl.de/) database [30]. The SMILES format of the identified compounds was uploaded to the STITCH and SEA databases with the “*Homo sapiens*” setting. Afterward, the target proteins corresponding to the compounds screened from the three target databases were standardized in the UniProt (https://www.uniprot.org) database, with the properties set to “reviewed” and “human” [31]. Finally, the targets from the three databases were merged, and the duplicated targets were removed. A Venn Diagram (http://bioinformatics.psb.ugent.be/webtools/Venn/) was used to filter the repeated targets among active components of herbs and to construct the herbs—ingredients—targets network using Cytoscape v.3.2.1 software.

### Target dataset for COVID-19

COVID-19-related targets were retrieved from the Human Gene Database (GeneCards, https://www.genecards.org/) [32] with the keyword “novel coronavirus pneumonia.” The targets were also sent to the UniProt Database for normalization.

### Protein‒protein interaction

To clarify the interaction between CDPF-related targets and COVID-19 targets, we screened for overlaps between CDPF-related targets and COVID-19 target-related targets. Then, we uploaded the list of overlapping proteins to the Search Tool for the Retrieval of Interacting Genes/Proteins database (STRING) v.11.0 (https://string-db.org/) for PPI analysis [33], with the species limited to “*Homo sapiens*” and the confidence score cut-off set at 0.4, and the rest of the settings were default. Finally, the final PPIs network was established.

### Network construction and analysis

We constructed a PPI network for active ingredients of CDPF and putative COVID-19-related targets of these ingredients based on STRING, and applied Cytoscape v.3.2.1 software to visualize and analyse the interaction network [34]. To study further into the network, the candidates were screened with clustering. Clustering with the Overlapping Neighborhood Expansion (ClusterONE) plug-in of the Cytoscape software was employed to mine the highly interacting gene modules [35], with the minimum size parameter set to 3. The cytoHubba plug-in of the Cytoscape software was used to obtain the top-10 genes, which fetches the shortest path among a group of nodes, based on a mixed character calculation (MCC) score [36].

### Gene ontology-enrichment and pathway-enrichment analyses

The candidate genes screened by ClusterONE were further analysed to understand their gene ontology (GO) function, by using the Cytoscape plug-in ClueGO to annotate and visualise the interrelations of terms and functional groups in biological networks. The relevant biological processes (BP), molecular functions (MF), cellular components (CC), and Kyoto Encyclopedia of Genes and Genomes (KEGG) pathway-enrichment analysis were selected, with a threshold value of *p* < 0.05 and a kappa score ≥ 0.4.

### Molecular docking

Through the above-mentioned cytoHubba analysis, the hub genes for the treatment of COVID-19 were obtained. The top hub gene and other key genes with the more connected active component in herbs were linked by molecular docking. The structural formula (SDF format) of the compounds were downloaded from the PubChem database and converted to PDB format with Open babel v.2.4.1 [37] from the RCSB Protein Data Bank (PDB, https://www.rcsb.org/) to obtain the crystal structure of the core target [38]. The targets were processed by removing water, adding hydrogen, optimising amino acids, and selecting the magnetic field, and the pdbqt format was saved as a pair acceptor. Atomic charges and assigned atom types were added to compounds in the PDB format, and the pdbqt format was saved as a docked ligand. The active site for molecular docking was determined and size was set. Finally, Autodock Vina v.1.1.2 was run to perform molecular docking [39]. PyMOL v.2.3 software [40], and Molecular Operating Environment v.2.2 (MOE) software [41] were used to visualize the docking results, and based on the binding conformations of the docking results of each compound, the docking results with lower binding energy and better conformation were selected.

## Results

### Active components screening for CDPF

In this study, components of 20 herbal medicines in CDPF were collected, of which Ma Huang (MH, *Herba Ephedrae)*, Ku Xing Ren (KXR, *Semen Armeniacae Amarum*), Qiang Huo (QH, *Rhizoma et Radix Notopterygii*), Ting Li Zi (TLZ, *Semen Descurainiae*), Guan Zhong (GZ, *Rhizoma Dryopteris Crassirhizomae)*, Xu Chang Qing (XCQ, *Radix Cynanchi Paniculati*), Guang Huo Xiang (GHX, *Herba Pogostemonis),* Pei Lan (PL, *Herba Eupatorii*), Cang Zhu (CZ, *Rhizoma Atractylodis*), Fu Ling (FL, *Poria*), Bai Zhu (BZ, *Rhizoma Atractylodis Macrocephalae*), and Hou Po (HP, *Cortex Magnoliae Officinalis*) were identified from the TCMSP database; Shi Gao (SG, *Gypsum Fibrosum*), Di Long (DL, *Lumbricus),* Jiao Bing Lang (JBL, *Semen Arecae*), Cao Guo (CG, *Fructus Tsaoko*), and Sheng Jiang (SJ, *Rhizoma Zingiberis Recens*) were retrieved from the TCMID database; and Jiao Shan Zha (JSZ, *Fructus Crataegi*) [42–44], Jiao Shen Qu (JSQ, *Massa Medicata Fermentata*) [44], and Jiao Mai Ya (JMY, *Fructus Hordei Germinatus*) [44–46] were obtained by literature mining. Next, according to the screening criteria of the ADME, including the OB (or GI absorption) and DL, 193 active ingredients of CDPF were retrieved after duplicated targets were eliminated. Details of component information are provided in the Additional file 1: Table S1.

### Target fishing for active CDPF components and construction of the component‒target network

The TCMSP, SEA, and STITCH databases were searched for candidate targets of active CDPF ingredients. After fishing for targets, 1172 targets were found for MH, 488 targets were found for KXR, 276 targets were found for QH, 647 targets were found for TLZ, 165 targets were found for GZ, 25 targets were found for XCQ, 397 targets were found for GHX, 211 targets were found for PL, 276 targets were found for CZ, 77 targets were found for FL, 73 targets were found for BZ, 43 targets were found for HP, 371 targets were found for CG, 165 targets were found for SJ, 6 targets were found for DL, 42 targets were found for JBL, 494 targets were found for JMY, 1023 targets were found for JSZ, and 85 targets were found for JSQ. Details of target information are provided in the Additional file 2: Table S2.

The main herbs of CDPF had synergistic effects with each other as exhibited by a Venn diagram. BZ, CG, CZ, FL, GZ, HP, GHX, JMY, JSZ, KXR, MH, PL, QH, SJ, TLZ, and XCQ shared three targets, CG, CZ, GZ, GHX, JMY, JSZ, KXR, MH, PL, QH, and TLZ shared seven targets, BZ, CG, CZ, FL, KXR, MH, PL, QH, SJ, and TLZ shared 12 targets, and CG, GHX, JMY, JSZ, MH, and TLZ shared up to 62 targets, etc. Although there were too many different lists to be captured in a single diagram, the details of the Venn diagram are provided in text form in the Additional file 3: Table S3.

**Fig. 1:**
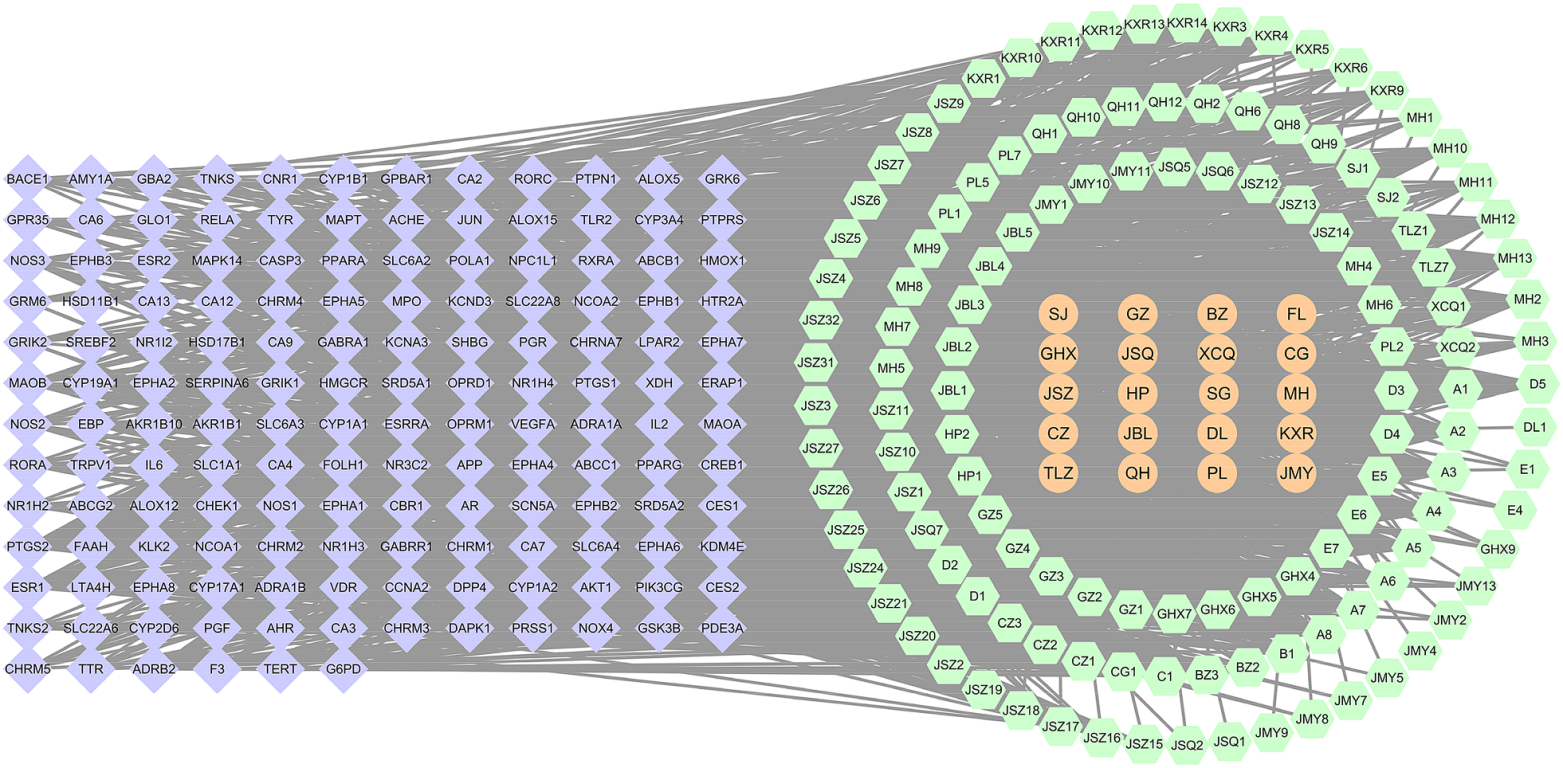
High connections network construction of “herbs—ingredients—targets”. High connections containing the nodes above the average degree in the whole “herbs—ingredients—targets” network. The “Ellipse” node represents the herb, the “Hexagon” node represents the ingredient, and the “Diamond” node represents the target

Overall, 949 candidate targets of active CDPF components were identified after removing duplication, and the herbs—ingredients—targets interaction network was consequently constructed using Cytoscape v.3.2.1 software. In order to simplify the network, the nodes above the average degree were selected to build the high connections of “herbs—ingredients—targets” network (Fig. 1). The network containing all the nodes of 949 candidate targets of active CDPF components was shown in Additional file 4: Fig. S1.

### Potential COVID-19 targets of CDPF

For disease target identification, 259 target genes that occur in COVID-19 were identified from the GeneCards database (Additional file 5: Table S4). Seventy-one target genes overlapped between CDPF and COVID-19 and were selected as potential targets for further analysis (Table 2).

**Table 2:** Potential targets of CDPF against COVID-19

Protein name	Gene symbol	Uniprot ID
Prostaglandin G/H synthase 1	PTGS1	P23219
Prostaglandin G/H synthase 2	PTGS2	P35354
Mothers against decapentaplegic homolog 3	SMAD3	P84022
Heme oxygenase 1	HMOX1	P09601
Granulocyte–macrophage colony-stimulating factor	CSF2	P04141
Nitric oxide synthase, inducible	NOS2	P35228
Peroxisome proliferator activated receptor gamma	PPARG	P37231
Phosphatidylinositol-4,5-bisphosphate 3-kinase catalytic subunit, gamma isoform	PIK3CG	P48736
Dipeptidyl peptidase IV	DPP4	P27487
Nitric-oxide synthase, endothelial	NOS3	P29474
Transcription factor p65	RELA	Q04206
Apoptosis regulator Bcl-2	BCL2	P10415
Apoptosis regulator BAX	BAX	Q07812
Tumor necrosis factor	TNF	P01375
Caspase-3	CASP3	P42574
Mitogen-activated protein kinase 8	MAPK8	P45983
Signal transducer and activator of transcription 1-alpha/beta	STAT1	P42224
Intercellular adhesion molecule 1	ICAM1	P05362
Transthyretin	TTR	P02766
Coagulation factor Xa	F10	P00742
Epidermal growth factor receptor	EGFR	P00533
Bcl-2-like protein 1	BCL2L1	Q07817
Proto-oncogene c-Fos	FOS	P01100
Mitogen-activated protein kinase 1	MAPK1	P28482
Interleukin-10	IL10	P22301
Retinoblastoma-associated protein	RB1	P06400
Interleukin-6	IL6	P05231
Cellular tumor antigen p53	TP53	P04637
Caspase-8	CASP8	Q14790
Superoxide dismutase [Cu–Zn]	SOD1	P00441
Protein kinase C alpha type	PRKCA	P17252
Interleukin-1 beta	IL1B	P01584
C–C motif chemokine 2	CCL2	P13500
Interleukin-8	CXCL8	P10145
Protein kinase C beta type	PRKCB	P05771
Heat shock protein beta-1	HSPB1	P04792
Interleukin-2	IL2	P60568
Plasminogen activator inhibitor 1	SERPINE1	P05121
Interferon gamma	IFNG	P01579
Interleukin-1 alpha	IL1A	P01583
Poly [ADP-ribose] polymerase 1	PARP1	P09874
C-X-C motif chemokine 11	CXCL11	O14625
C-X-C motif chemokine 2	CXCL2	P19875
C-X-C motif chemokine 10	CXCL10	P02778
CD40 ligand	CD40LG	P29965
Phosphatidylinositol 3-kinase regulatory subunit alpha	PIK3R1	P27986
Induced myeloid leukemia cell differentiation protein Mcl-1	MCL1	Q07820
Interleukin-4	IL4	P05112
Cyclic AMP-responsive element-binding protein 1	CREB1	P16220
Glucose-6-phosphate 1-dehydrogenase	G6PD	P11413
Catalase	CAT	P04040
Cytosolic phospholipase A2	PLA2G4A	P47712
Protein kinase C epsilon type	PRKCE	Q02156
Mitogen-activated protein kinase 3	MAPK3	P27361
Calmodulin-1	CALM1	P0DP23
T-cell surface glycoprotein CD4	CD4	P01730
Mitogen-activated protein kinase 14	MAPK14	Q16539
Nuclear factor NF-kappa-B p105 subunit	NFKB1	P19838
Angiotensin-converting enzyme	ACE	P12821
Cyclin-dependent kinase 4	CDK4	P11802
CD81 antigen	CD81	P60033
Eukaryotic translation initiation factor 2 subunit 1	EIF2S1	P05198
Growth factor receptor-bound protein 2	GRB2	P62993
Apolipoprotein E	APOE	P02649
C–C chemokine receptor type 3	CCR3	P51677
Angiotensin-converting enzyme 2	ACE2	Q9BYF1
Aminopeptidase N	ANPEP	P15144
Serine/threonine-protein kinase/endoribonuclease IRE1	ERN1	O75460
Cyclic AMP-dependent transcription factor ATF-2	ATF2	P15336
Cathepsin B	CTSB	P07858
Peptidyl-prolyl cis–trans isomerase A	PPIA	P62937

### PPI network analysis

The STRING database was used to acquire PPI relationships of potential protein targets of CDPF as related to the treatment of COVID-19. By using these targets, the network of PPI relationships was shown to contain 71 nodes (representing active proteins) and 1006 edges (representing the interaction between the active proteins and proteins), with an average node degree of 28.3 and a PPI-enrichment *p* value of < 1.0e−16.

**Fig. 2:**
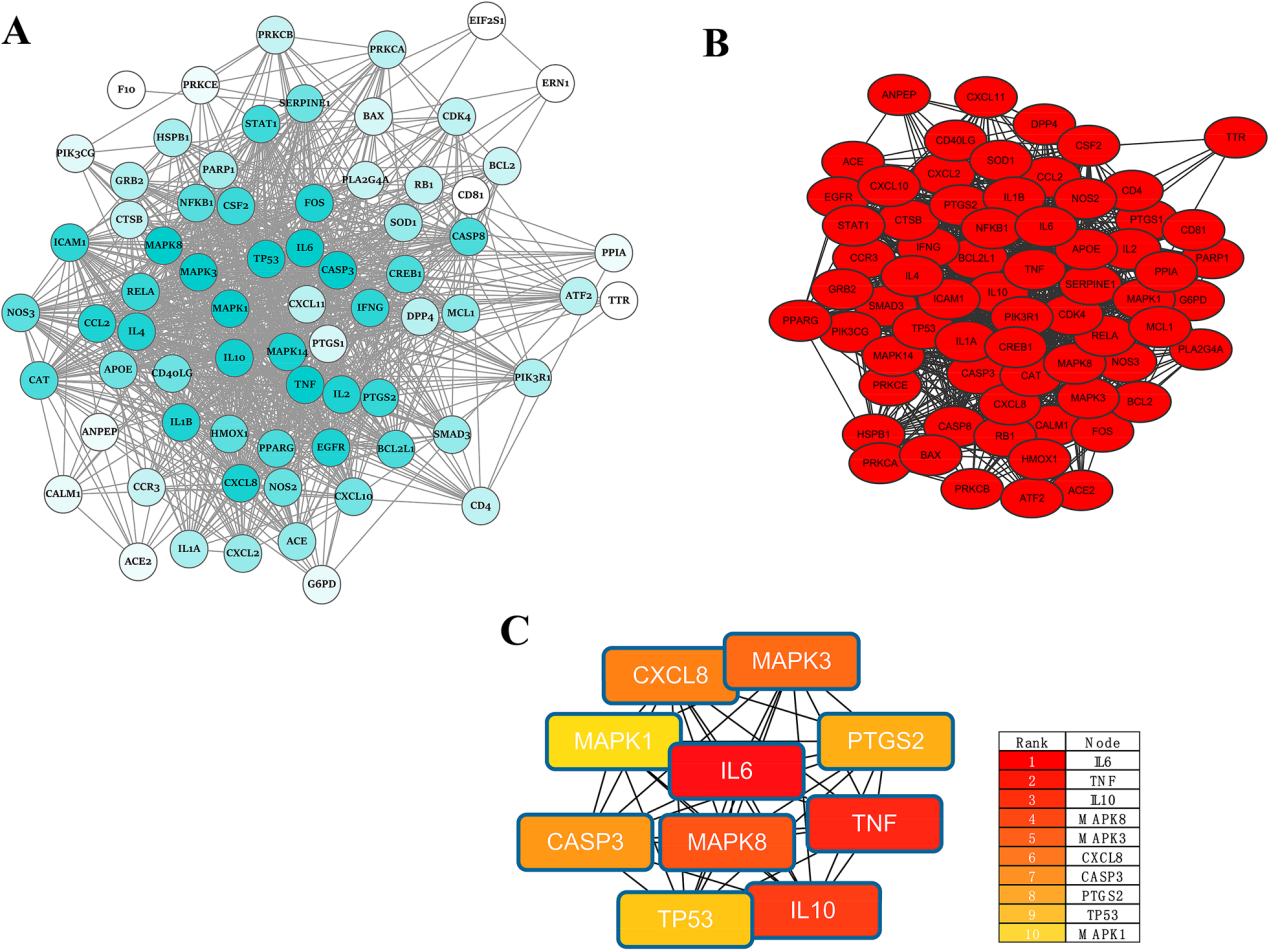
Protein-protein interaction (PPI) Analysis. A. PPI networks of all candidate targets of CDPF for the treatment of COVID-19 from STRING 11.0 and was exhibited by Cytoscape plug-in. Nodes represent proteins (Low values to bright colors depend on the degree). Edges represent protein–protein associations. B. The most significant module identified by ClusterONE plug-in (Density = 0.438, Quality = 0.874, *p *< 0.001). C. The top 10 targets (hub targets) in the PPI network ranked by maximal clique centrality (MCC) using cytoHubba plug-in

In the PPI network, constructed with the Cytoscape software using parameters such as a minimum required interaction score > 0.4) (Fig. 2a), the most significant module (Density = 0.438, Quality = 0.874, *p* < 0.001) containing 68 nodes was then recognized by ClusterONE (Fig. 2b).

Moreover, the top 10 targets ranked by the MCC method were identified as hub genes using the cytoHubba plug-in (Fig. 2c). These targets included interleukin-6 (*IL6*), tumour necrosis factor (*TNF*), interleukin-10 (*IL10*), mitogen-activated protein kinase 8 (*MAPK8*), mitogen-activated protein kinase 3 (*MAPK3*), interleukin-8 (*CXCL8*), caspase-3 (*CASP3*), prostaglandin G/H synthase 2 (*PTGS2*), cellular tumour antigen p53 (*TP53*), and mitogen-activated protein kinase 1 (*MAPK1*).

### GO-enrichment analysis

**Fig. 3:**
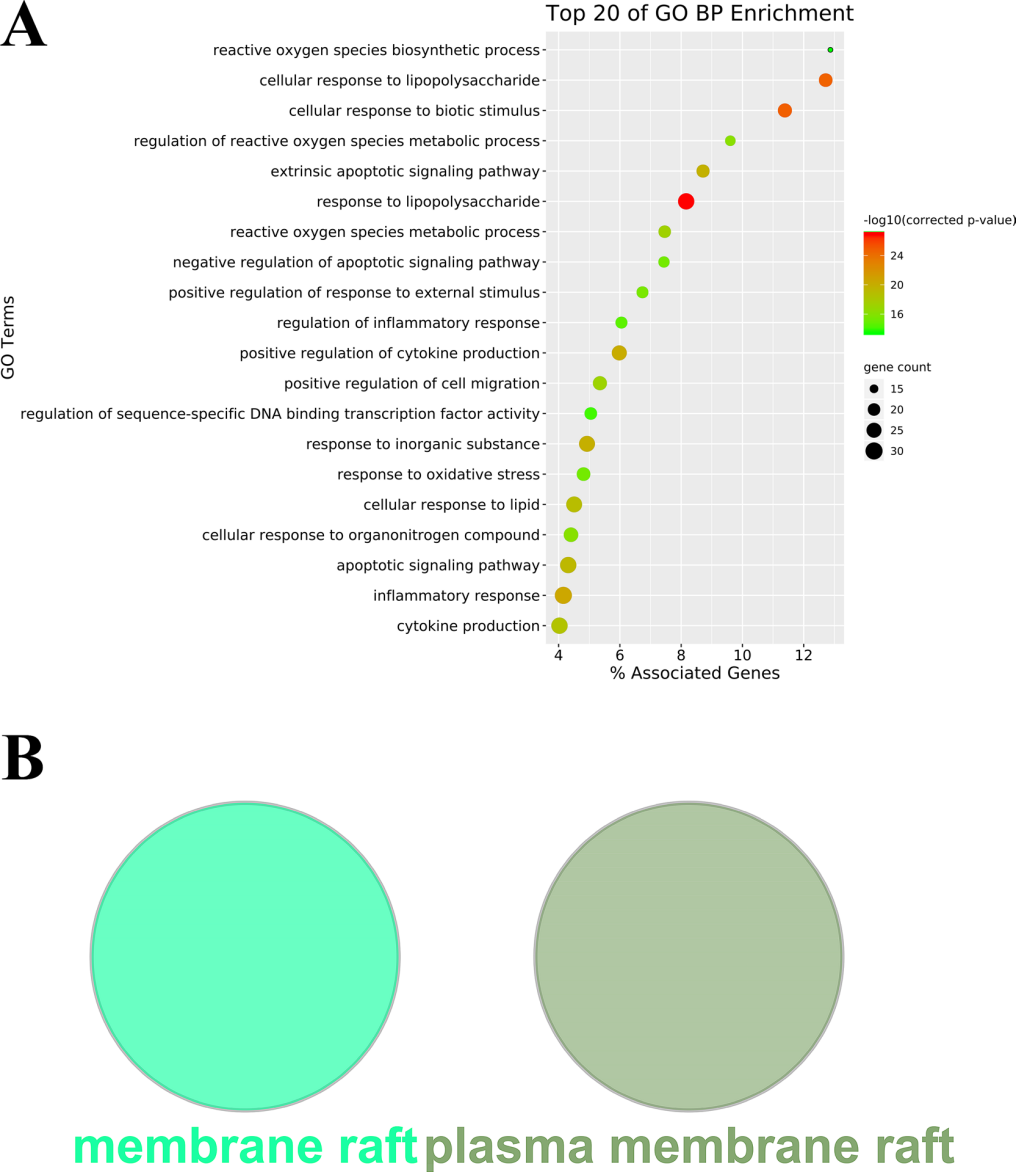

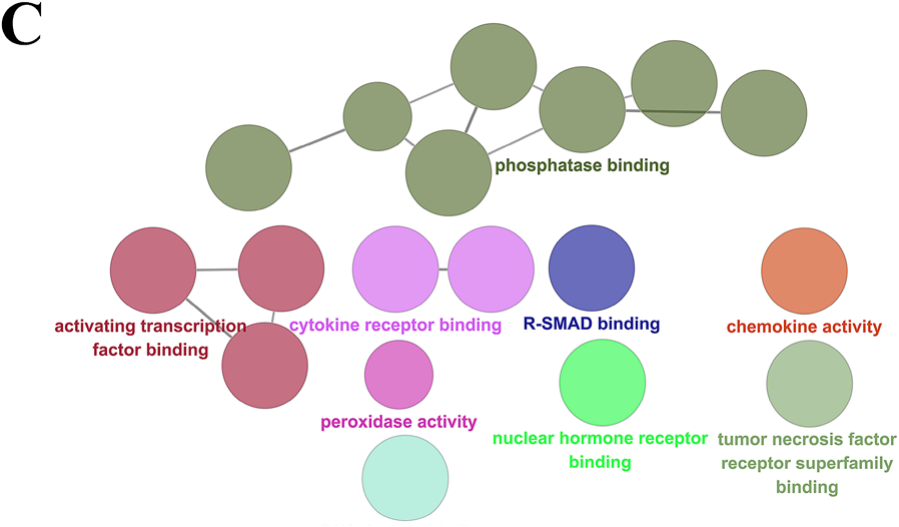
GO enrichment analysis of the potential targets of CDPF against COVID-19 by ClueGO plug-in. **a** Top 20 GO terms of biological process (BP) were listed by bubble chart according to “term *p*-value corrected with Bonferroni step down”; GO terms, including **b** cellular component (CC) terms, **c** molecular function (MF) terms, are represented as nodes, and size of the node represents significance. Only the most significant terms in the group have been labelled

To determine the biological features of the candidate targets screened by ClusterONE, GO analysis was accomplished by the Cytoscape plug-in ClueGO. Based on filter conditions of *p* < 0.05 and kappa score ≥ 0.4, GO function-enrichment analysis resulted in 466 items, of which BP accounted for 446, CC for 2, and MF for 18 items. BP analysis revealed that the candidate targets were markedly enriched for cellular response to biotic stimulus, inflammatory response, regulation of inflammatory response, reactive oxygen species metabolic process, positive regulation of response to external stimulus, cellular response to external stimulus, cytokine production, regulation of sequence-specific DNA binding transcription factor activity, regulation of chemokine production, reactive oxygen species biosynthetic process, regulation of leukocyte migration, leukocyte homeostasis, B cell proliferation, T cell migration, lymphocyte activation involved in immune response, regulation of nitric oxide biosynthetic process, and response to reactive oxygen species, positive regulation of hemopoiesis, regulation of vasculature development, platelet activation, regulation of blood vessel diameter, positive regulation of cytokine production, regulation of cytokine biosynthetic process, response to extracellular stimulus, response to drug, response to toxic substance, regulation of chemokine biosynthetic process, regulation of production of molecular mediator of immune response, acute inflammatory response, regulation of acute inflammatory response, regulation of immunoglobulin production, receptor-mediated virion attachment to host cell, cellular oxidant detoxification, defense response to protozoan, positive regulation of homeostatic process, and other (Additional file 6: Table S5-1). Here top 20 GO terms of BP were listed by bubble chart according to “term *p*-value corrected with Bonferroni step down”. The CC analysis showed that these candidate genes were enriched in membrane rafts and plasma membrane rafts (Fig. 3b, Additional file 6: Table S5-2). Changes in the MF of the targets were mainly significantly enriched for cytokine receptor binding, phosphatase binding, BH3 domain binding, activating transcription factor binding, chemokine activity, tumour necrosis factor receptor superfamily binding, nuclear hormone receptor binding, R-SMAD binding, and peroxidase activity (Fig. 3c, Additional file 6: Table S5-3).

### KEGG pathway analysis

**Fig. 4:**
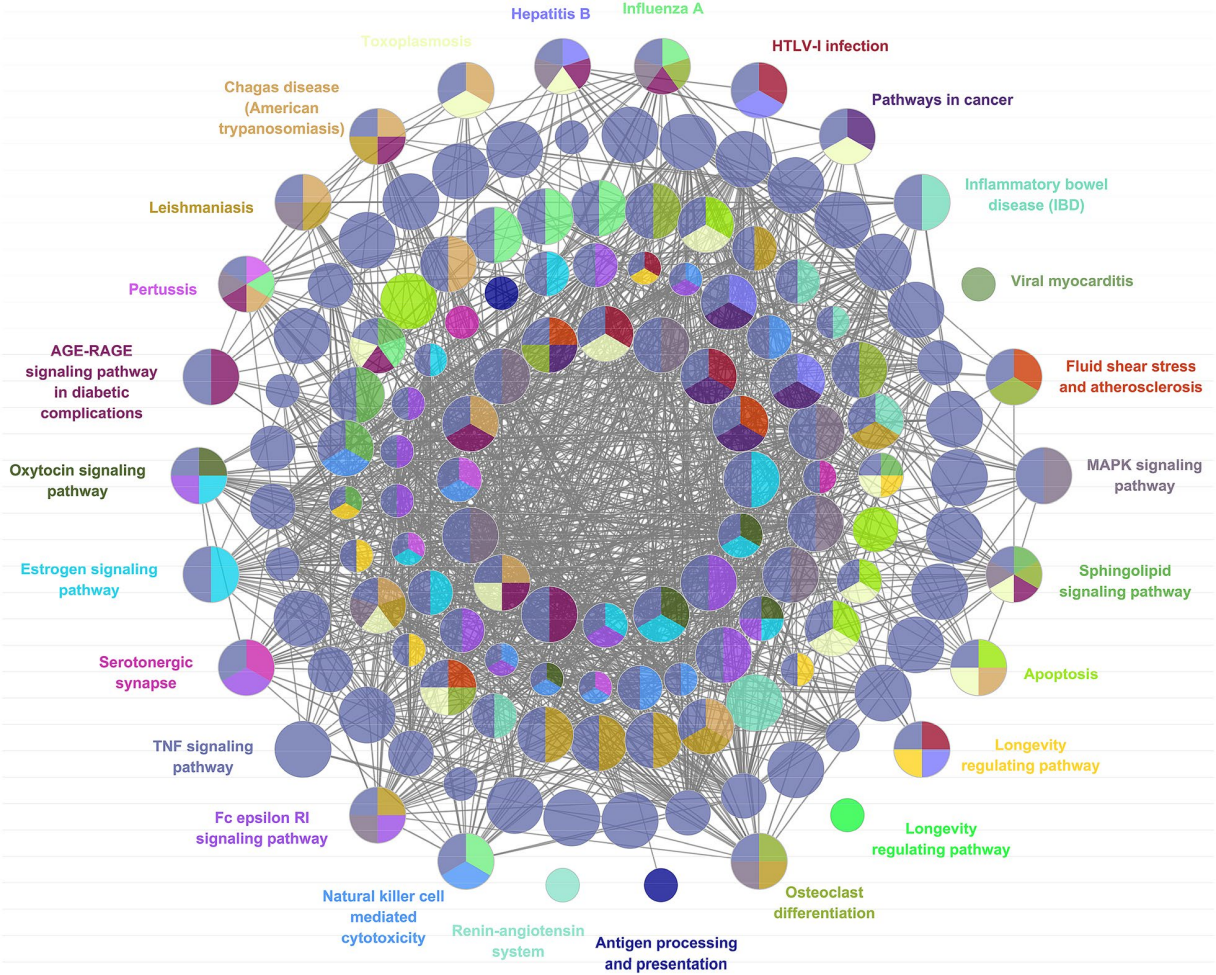
KEGG pathway analysis of the potential targets of CDPF against COVID-19 by ClueGO plug-in. KEGG terms are represented as nodes, only the most significant terms in the group have been labelled, and small labels are hidden

To explore the potential mechanism related to these candidate targets identified by ClusterONE, KEGG pathway analysis was performed. Based on filter conditions of *p* < 0.05 and kappa score ≥ 0.4, KEGG pathway analysis returned 137 items, including TNF signaling pathway, MAPK signaling pathway, Fc epsilon RI signaling pathway, toxoplasmosis, leishmaniasis, estrogen signaling pathway, natural killer cell mediated cytotoxicity, chagas disease, osteoclast differentiation, longevity regulating pathway, influenza A, sphingolipid signaling pathway, inflammatory bowel disease (IBD), apoptosis, hepatitis B, HTLV-I infection, pertussis, antigen processing and presentation, renin-angiotensin system, and viral myocarditis, AGE-RAGE signaling pathway in diabetic complications, and so on (Fig. 4, Additional file 7: Table S6). These items revealed that the potential pathways affected by CDPF mainly involved improving immunity, anti-inflammatory effects, fighting against viruses and other pathogens.

### Molecular docking

**Fig. 5:**
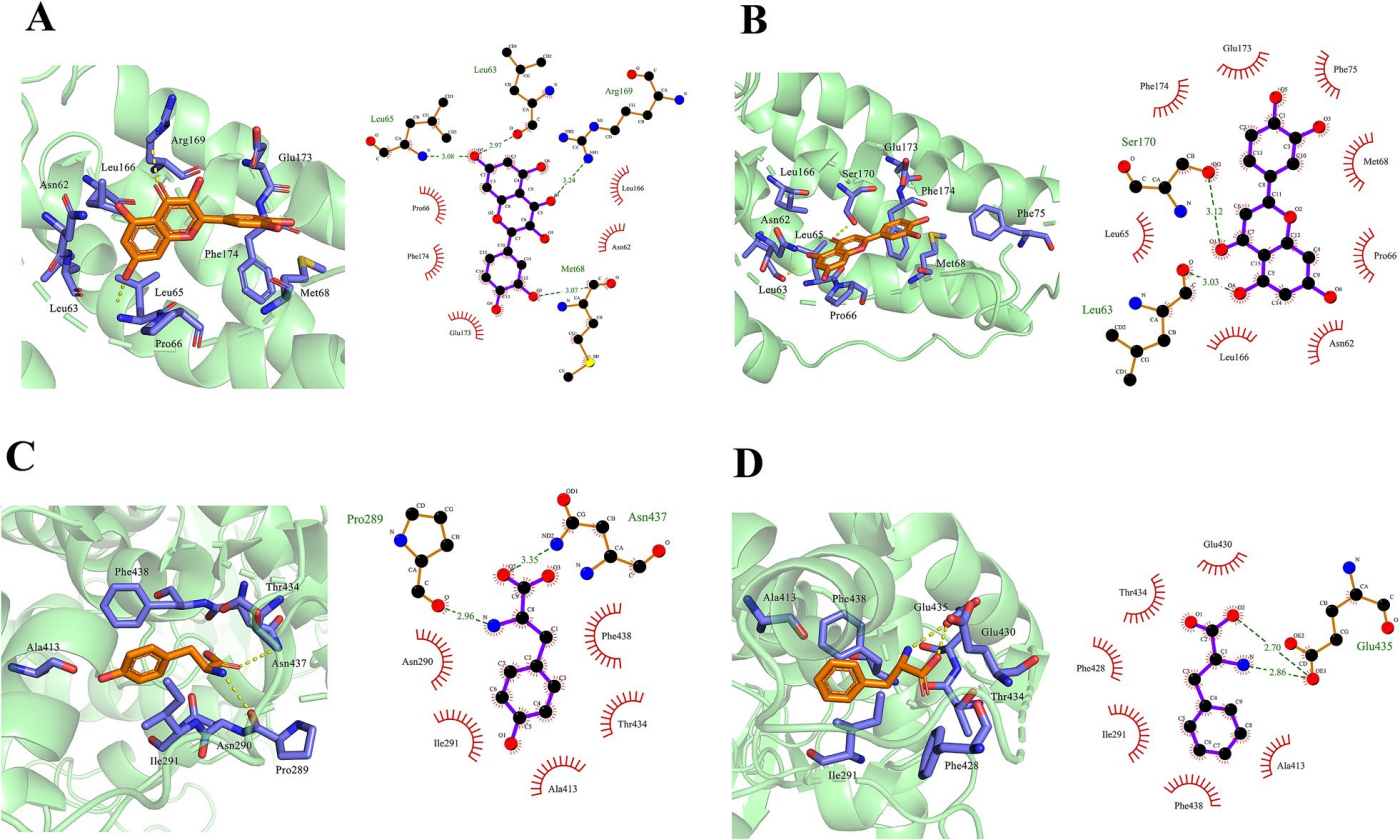
Molecular docking diagram. Molecular models of the binding of IL6 with **a** quercetin and **b** luteolin, ACE2 with **c**l-Tyrosine and **d**l-Phenylalanine shown as 3D diagrams and 2D diagrams

Molecular simulation was used to verify the binding ability of CDPF bioactive components to the key targets and explore their accurate binding modes. In this respect, the top hub target, IL6 (PDB ID: 1IL6), was selected, using quercetin and luteolin as its ligands; and as a typical receptor for SARS-CoV-2 entry into host cells, ACE2 (PDB ID: 3D0G) was chosen, using l-tyrosine and l-phenylalanine as its ligands. In this analysis, the value of the vina score indicates the binding activity between a compound and a protein. The more negative the vina score, the more stably the compound binds to the protein. As shown in Fig. 5, IL6 demonstrates strong binding to quercetin (Score = − 7.3) and luteolin (Score = − 7.2); similarly, ACE2 binds strongly to l-tyrosine (Score = − 6.5) and l-phenylalanine (Score = − 6.3). In terms of interaction points, IL6 mainly interacted with quercetin via amino acid residues Asn62, Leu166, Arg169, Glu173, Leu63, Leu65, Pro66, Phe174, and Met68, and with luteolin via residues Leu166, Ser170, Glu173, Phe174, Phe75, Asn62, Leu63, Leu65, Pro66, and Met68. ACE2 mainly interacted with l-tyrosine via amino acid residues Ala413, Phe438, Thr434, Asn437, Ile291, Asn290, and Pro289; and with l-phenylalanine via residues Ala413, Phe438, Glu435, Glu430, Ile291, Phe428, and Thr434.

## Discussion

TCM prescriptions are rich in resource-based chemical components, with complex chemical structures, different targets, and diverse action pathways, which work together to exert their unique clinical efficacy. In order to explain the therapeutic basis of CDPF against COVID-19 scientifically, this study revealed the active ingredients and potential molecular mechanism by which CDPF treatment is effective against COVID-19, and provides a reference basis for the wider application and further mechanistic investigations of CDPF in the fight against COVID-19.

First, we considered the compound’s feasibility for use as a drug, based on the database of TCMSP and TCMID and related references, combining the OB (or GI absorption) and DL values of the compound. Consequently, 193 active compounds of CDPF were assessed and 949 candidate targets of these active compounds were screened through drug target-prediction databases. Venn plots of these candidate targets showed that several herbs shared common targets; for example, the CG, GHX, JMY, JSZ, MH, and TLZ shared up to 62 targets, illustrating the synergy between herbs. We thus constructed an herbs—ingredients—targets network, which intuitively reflected the characteristics of CDPF, which has multiple components and targets.

We retrieved 259 candidate COVID-19 targets from the GeneCards database. Through target mapping, we found 71 targets in common between CDPF active component-targets and these disease targets, which were considered potential targets for CDPF in the treatment of COVID-19. To identify functional connections among these common targets, PPIs were predicted using STRING. We carried out module analysis on a PPI network via the ClusterONE plug-in in Cytoscape; modularization is an important step, as it helps to reduce the noise of the data. By modularization, the most significant cluster (Density = 0.438, Quality = 0.874, p < 0.001) was obtained, which contained 68 seed proteins. We subsequently performed GO and KEGG analyses of these seed proteins.

Using ClueGo enrichment analysis, significant biological processes related to the anti-COVID-19 effects of CDPF were identified. These mainly focused on four areas: (1) Cellular responses to external stimuli, such as cellular response to external stimulus, positive regulation of response to external stimulus, response to extracellular stimulus, cellular response to biotic stimulus, and even receptor-mediated virion attachment to host cell. (2) Regulation of blood production and circulation, such as regulation of vasculature development, platelet activation, positive regulation of hemopoiesis, and regulation of blood vessel diameter. SARS-CoV-2 may cause blood clotting and trigger the risk of stroke [47–49]; the above biological processes may be effective against the occurrence of blood clotting. (3) Free radical regulation, such as reactive oxygen species metabolic process, reactive oxygen species biosynthetic process, regulation of nitric oxide biosynthetic process, and response to reactive oxygen species. Some studies have indicated that high levels of reactive oxygen species and other free radicals are generated after virus invasion, thereby inducing damage to the organism [50–52]. These biological functions involve the regulation of free radical production and metabolism. (4) Immune regulation and anti-inflammatory effects, such as cytokine production, regulation of cytokine biosynthetic process, regulation of inflammatory response, regulation of leukocyte migration, regulation of cytokine biosynthetic process, regulation of production of molecular mediator of immune response, regulation of leukocyte migration, leukocyte homeostasis, B cell proliferation, T cell migration, lymphocyte activation involved in immune response, regulation of chemokine production, regulation of acute inflammatory response, regulation of immunoglobulin production, positive regulation of lymphocyte proliferation. In order to fight against a viral invasion, the organism stimulates a series of immune responses. Leukocytes quickly migrate to the site of the virus invasion to surround and phagocytize the virus [53, 54]. As the virus continues to replicate, B lymphocytes are activated by antigens, forming plasma cells, and producing immunoglobulins (antibodies), and then antigen–antibody binding participates in the humoral immune response through the blood and body fluids [55, 56]. Moreover, a large number of cytokines are released by immune cells to play an anti-viral role; these include ILs, interferons, TNFs, colony stimulating factors, chemokines, and growth factors [57–59]. However, while the body produces an immune response to eliminate the virus, the cytokines secreted by the immune cells activate further immune cells, which in turn produce more cytokines, and induce a “cytokine storm.” Eventually, the immune system begins to attack the normal cells of the host [60, 61]. This reflects two-way regulation: on the one hand, the body can quickly produce an immune response to play a role in eliminating the virus; on the other hand, it can regulate the production and secretion of cytokines to avoid the occurrence of a cytokine storm, which would cause damage to the organism.

KEGG pathway analysis showed that CDPF mainly regulates disease pathways induced by viruses, such as hepatitis B, influenza A, HTLV-I infection, and viral myocarditis. Additionally, CDPF regulates pathways of diseases caused by external organisms, such as bacteria or worms, including pertussis, leishmaniasis, chagas disease, and toxoplasmosis, etc. Immune and inflammatory pathways, such as antigen processing and presentation, natural killer cell mediated cytotoxicity [62], Fc epsilon RI signaling pathway, associating with the secretion of cytokines, such as TNF-α, contributing to inflammatory responses [63], TNF signaling pathway [64], and MAPK signaling pathway [65], and inflammatory disease-related pathways, such as IBD, are also involved. Sphingolipid signaling pathway utilizes second messenger functions involved in a variety of cellular signalling pathways; sphingolipids appear to be mediators of inflammation and are novel therapeutic targets in inflammatory disease [66]. CDPF also targeted the AGE-RAGE signaling pathway, which is involved in diabetic complications. Diabetes mellitus is a major comorbidity complicating COVID-19, and the association between glycaemic control and prognosis in patients with comorbid COVID-19 and diabetes has been reported [67]. ACE2, a gene involved in the renin-angiotensin system, has been shown to be a therapeutic target in COVID-19, as SARS-CoV-2 uses ACE2 as a receptor to enter host cells [68, 69]. In addition, pathways related to apoptosis, longevity, and estrogen signalling are also regulated by CDPF.

Moreover, in the PPI network, the top 10 targets (IL6, TNF, IL10, MAPK8, MAPK3, CXCL8, CASP3, PTGS2, TP53, and MAPK1) were regarded as hub genes that may play important roles in the treatment of COVID-19 by CDPF and are involved in the regulation of immunity and inflammation. For example, TNF has immune regulatory, proinflammatory [70], and anti-viral functions [71], MAPK8 modulates lymphocyte homeostasis [72], IL10 is generally considered to be an anti-inflammatory cytokine [73, 74], but production of IL-10 has also been shown to be detrimental during high-dose primary influenza challenge [75]. CXCL8 increases recruitment of principal human neutrophils and is a major inflammatory mediator [76], while PTGS2 is also an inflammatory marker [77]. The most notable factor was IL6, which plays a key role in the cytokine storm [78, 79], and is used as a clinical early warning index in the diagnosis and treatment of COVID-19 [80, 81]. IL6 plays a central role in the acute inflammatory response, and a long duration of its release can also be used to assess the severity of infection and judge prognosis [82, 83]. Dynamic observation of IL-6 levels can assist in understanding the progression of infectious diseases and the response to treatment [84, 85].

We then performed docking studies for IL6, using the critical ingredients quercetin and luteolin as ligands, and for ACE2, the receptor by which SARS-CoV-2 typically enters host cells, with its ligands, l-tyrosine and l-phenylalanine. Molecular docking allows assessment of whether it is thermodynamically possible for the ligand and protein to bind. The binding energies of these docking results further helped to refine the targets of CDPF. Finally, the vina scores of the active compounds with the key targets were all negative and less than −6, and their interaction points were revealed, and demonstrated that quercetin and luteolin, and l-tyrosine and l-phenylalanine had good binding activities to IL6 and ACE2, respectively.

## Conclusions

**Fig. 6:**
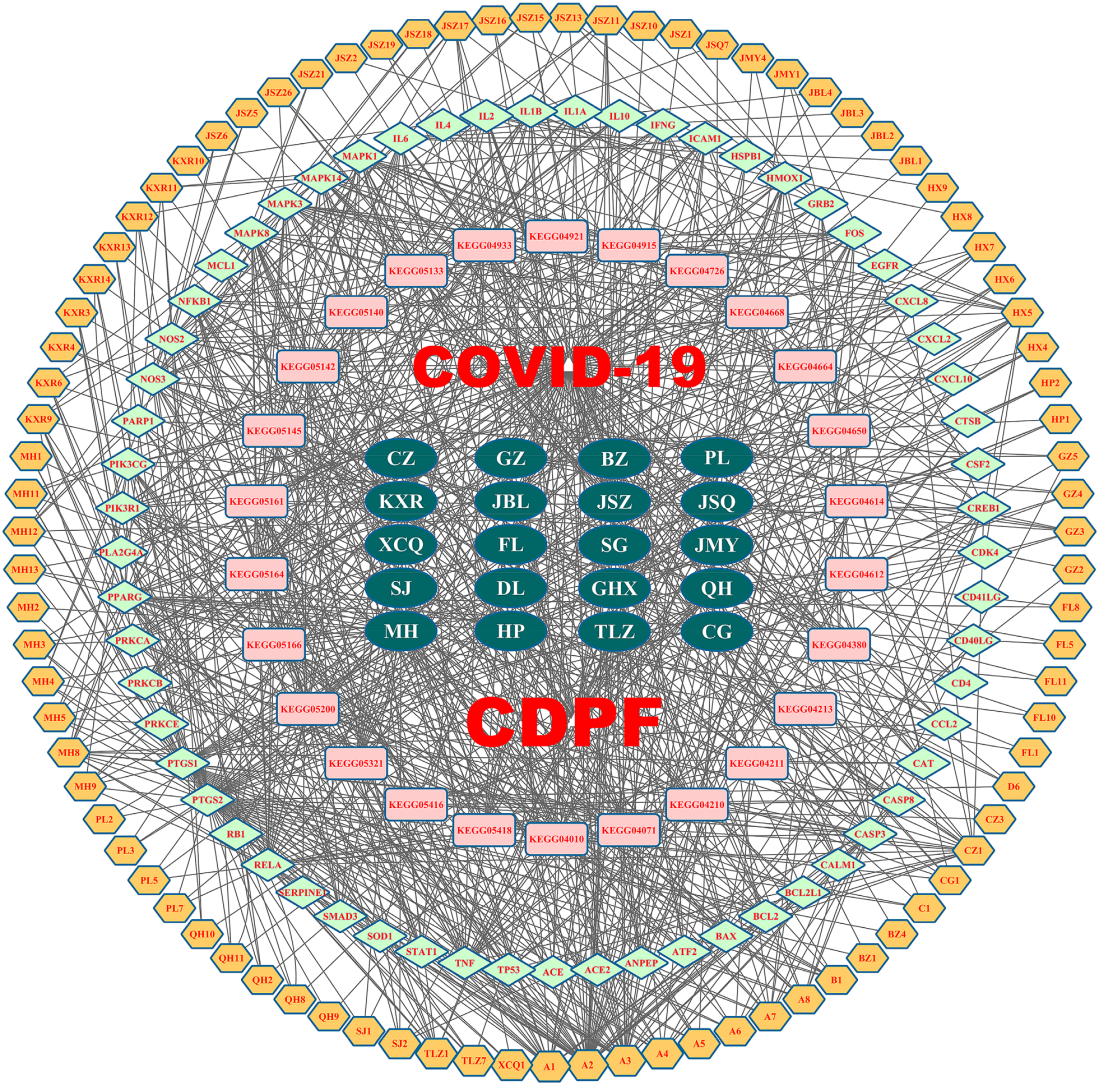
Network construction of “CDPF—herbs—ingredients—targets—pathways—COVID-19”. The “Ellipse” node represents the herb, the “Hexagon” node represents the ingredient, the “Diamond” node represents the target, the “Rectangle” node represents the pathway, and the small white “Triangle” node with big letters represents CDPF and COVID-19

In this study, we systematically analysed the potential mechanism by which CDPF is effective in the treatment of COVID-19, based on network pharmacology. A CDPF—herbs—ingredients—targets—pathways—COVID-19 network was constructed (Fig. 6). The results demonstrated a synergistic effect between herbs and illustrated that CDPF could play pharmacological roles in the treatment of COVID-19 through multi component—multi target—multi pathway effects at the molecular level, mainly involving anti-viral, immune-regulatory, and anti-inflammatory actions. These findings may offer a reference basis for further investigations of the mechanism by which CDPF exerts effects against COVID-19.

## Supplementary information

**Additional file 1: Table S1.** The main active Components in CDPF.

**Additional file 2: Table S2.** Candidate targets of active ingredients in the CDPF.

**Additional file 3: Table S3.** The text results details of the Venn diagram.

**Additional file 4: Fig. S1.** Network of “herbs—ingredients—targets”. The “Ellipse” node represents the herb, the “Hexagon” node represents the ingredient, and the “Diamond” node represents the target.

**Additional file 5: Table S4.** Candidate targets of COVID-19.

**Additional file 6: Table S5.** 1: Node attribute information of GO analysis-biological process (BP). 2: Node attribute information of GO analysis-cellular component (CC). 3: Node attribute information of GO analysis-molecular function (MF).

**Additional file 7: Table S6.** Node attribute information of KEGG analysis.

## Data Availability

The datasets used and/or analysed during the current study are available from the corresponding author on reasonable request.

## Abbreviations

TCM: Traditional Chinese medicine
CDPF: Cold-Damp Plague Formula
COVID-19: Coronavirus disease 2019
SARS-CoV-2: Severe acute respiratory syndrome coronavirus 2
NCP: Novel coronavirus pneumonia
WHO: World Health Organization
TCMSP: Traditional Chinese Medicine Systems Pharmacology
TCMID: Traditional Chinese Medicines Integrated Database
OB: Oral bioavailability
DL: Drug likeness
SEA: Similarity ensemble approach
PPI: Protein–protein interaction
STRING: Search Tool for the Retrieval of Interacting Genes/Proteins database
ClusterONE: Clustering with Overlapping Neighborhood Expansion
MCC: Mixed character calculation
GO: Gene Ontology
BP: Biological processes
MF: Molecular functions
CC: Cellular components
KEGG: Kyoto Encyclopedia of Genes and Genomes
PDB: Protein Data Bank
